# Sequence-Specific Dimerization of a Transmembrane Helix in Amphipol A8-35

**DOI:** 10.1371/journal.pone.0110970

**Published:** 2014-10-27

**Authors:** Michael Stangl, Sebastian Unger, Sandro Keller, Dirk Schneider

**Affiliations:** 1 Department of Pharmacy and Biochemistry, Johannes-Gutenberg-University, Mainz, Germany; 2 Molecular Biophysics, University of Kaiserslautern, Kaiserslautern, Germany; Martin-Luther-Universität Halle-Wittenberg, Germany

## Abstract

As traditional detergents might destabilize or even denature membrane proteins, amphiphilic polymers have moved into the focus of membrane-protein research in recent years. Thus far, Amphipols are the best studied amphiphilic copolymers, having a hydrophilic backbone with short hydrophobic chains. However, since stabilizing as well as destabilizing effects of the Amphipol belt on the structure of membrane proteins have been described, we systematically analyze the impact of the most commonly used Amphipol A8-35 on the structure and stability of a well-defined transmembrane protein model, the glycophorin A transmembrane helix dimer. Amphipols are not able to directly extract proteins from their native membranes, and detergents are typically replaced by Amphipols only after protein extraction from membranes. As Amphipols form mixed micelles with detergents, a better understanding of Amphipol-detergent interactions is required. Therefore, we analyze the interaction of A8-35 with the anionic detergent sodium dodecyl sulfate and describe the impact of the mixed-micelle-like system on the stability of a transmembrane helix dimer. As A8-35 may highly stabilize and thereby rigidify a transmembrane protein structure, modest destabilization by controlled addition of detergents and formation of mixed micellar systems might be helpful to preserve the function of a membrane protein in Amphipol environments.

## Introduction

Traditionally, detergents are used to solubilize membrane proteins (MPs) for subsequent purification and *in vitro* analyses. However, as detergents might destabilize or even denature MPs [1], amphiphilic polymers have been introduced as a new class of surfactants suitable for keeping membrane proteins soluble in aqueous solution [2]-[11]. Amphipols (APols) are such amphiphilic copolymers (terpolymers), possessing a hydrophilic backbone and short hydrophobic chains. The thus far most common and best studied APol A8-35 shows high solubility in water at pH>7.0 and assembles into tetrameric nanoparticles, which together carry 75–80 octyl chains [2], [3], [5], [12]. Similar to detergents, APols do not aggregate until a particular concentration is reached. However, in contrast to most detergents, the critical aggregation concentration (CAC) is in the nanomolar range, allowing APols to form aggregates and stabilize MPs at very low concentrations. However, APols are not able to extract MPs from their native membranes, and thus, MPs are typically still extracted by classical detergents. Only in a subsequent step is the detergent substituted by an APol. Therefore, when working with APols, detergents are still vital. When detergents are added to APols at concentrations below their critical micellar concentration (CMC), APols and detergents form mixed micelles/aggregates [13]. In mixtures of non-ionic detergents with APols, the fraction of detergent molecules in the mixed micelles increases according to a near-ideal mixing behavior as the concentration of free detergent molecules in solution increases [13], [14].

Due to their low CAC values and their ability to render MPs soluble in aqueous solutions at very low bulk concentrations, APols are highly attractive for applications in MP research. A low APol-to-protein ratio implies that only few APol molecules are needed to provide a hydrophobic environment for MPs, which reduces the size of the hydrophobic sink when compared to classical detergents [10]. This might explain the less denaturing properties of APols on MPs [3]. Consequently, APols represent a class of membrane-mimetic surfactants that potentially preserve the structure as well as the function of MPs better than many detergents. In fact, more than 30 MPs have already been shown to form water-soluble complexes with APols [6], [15]. Bringing the MPs with APols in solution, is one fundamental step but the MPs also have to fold into their native and active conformation. Most MPs seem to still function after being trapped in APol particles [3]. As an example, bacteriorhodopsin correctly folds in A8-35 and accomplishes its entire photocycle, though with slower kinetics compared to detergents [10]. On the other hand, the sarcoplasmic Ca^2+^-ATPase can be solubilized in A8-35, but it shows very slow hydrolytic activity and Ca^2+^ dissociation [16]. The transmembrane (TM) region of the sarcoplasmic Ca^2+^-ATPase undergoes large conformational changes during its active process, which seems to be constrained by the tight, multiple A8-35 attachment [17].

As the formation of higher-ordered oligomeric MP structures depends on multiple specific TM helix-helix contacts, analyzing the stability of a sequence-specific TM helix dimer can be helpful in properly elucidating the impact of APols on MP structure and stability.

In recent years, association of TM α-helices has been studied to a great extent using the TM region of the human glycophorin A (GpA) protein, which forms a stable TM helix dimer. Sequence-specific GpA dimerization is mediated by the LIxxGVxxGVxT amino acid motif [18]-[20]. Especially the GxxxG motif promotes tight packing of two adjacent TM α-helices, resulting in Van der Waals packing interactions and formation of C_α_ hydrogen bonds [21], [22]. In fact, in the last two decades the GpA TM helix dimer became a paradigm for studying sequence-specificity in TM helix dimerization. GpA dimerization has been analyzed in various detergents and lipids [23]-[28], and the results have indicated that GpA TM helix association is driven by enthalpic and entropic forces [27]. Furthermore, detergent properties, such as head group chemistry, chain length, aggregation number, as well as the concentration of a particular detergent or phospholipid affect the stability of the GpA TM helix dimer [23]-[28]. Thus, the GpA TM helix can serve as a valuable probe to quantitatively determine the effect of an APol environment on the structure and stability of a sequence-specific TM helix–helix interaction.

## Materials and Methods

### Materials

Peptides corresponding to residues 69–101 of the human GpA TM domain (SEPEITLIIFGVMAGVIGTILLISYGIRRLIKK) were custom-synthesized and labeled at the N-terminus with either the donor or the acceptor dyes fluorescein (FL) and 5-6-carboxyrhodamine (TAMRA), respectively (Peptide Specialty Laboratories, Heidelberg, Germany). The purity of the labelled peptides was confirmed by high-performance liquid chromatography (HPLC) and mass spectrometry [24]. Based on this, the labeled peptides used in this study were>95% pure. Peptides were dissolved in 2,2,2-trifluoroethanol purchased from Sigma-Aldrich (Munich, Germany). APol A8-35 was purchased from Affymetrix (Santa Clara, USA) and sodium dodecyl sulfate (SDS) from Roth (Karlsruhe, Germany). *N*-dodecyl-β-D-maltopyranoside (DDM) was obtained from Sigma-Aldrich (Munich, Germany).

### FRET measurements

For Förster resonance energy transfer (FRET) measurements, equal concentrations of FL- and TAMRA-labeled GpA TM domains were used. Concentrations of the peptide stock solutions were determined from absorbance measurements on a Perkin Elmer Lambda 35 UV/VIS spectrophotometer. In all experiments, we used 0.25 µM for each of the two labeled GpA peptides. Peptides dissolved in TFE and A8-35 dissolved in ethanol were mixed, and organic solvents were removed under a gentle stream of nitrogen gas. Residual solvent traces were removed by vacuum desiccation overnight. The dried peptide/polymer film was then hydrated in 10 mM HEPES buffer (pH 7.4) containing 150 mM NaCl.

After at least 2 h of incubation at 37°C and 10 min of centrifugation at 16,000 *g*, steady-state fluorescence measurements were performed with the supernatant at 25°C on a Horiba Fluoromax 4 system with both excitation and emission slits at 3 nm. The excitation wavelength was set at 439 nm, and emission spectra were recorded from 480 to 650 nm. For FRET measurements with SDS, several concentrations of SDS, both below and above the CMC determined for the given buffer conditions, were used, and hydrated samples were incubated overnight.

Energy transfer *E* was calculated using the donor fluorescence intensities at 525 nm in presence and absence of acceptor according to
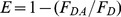
(1)


*F_D_* is the fluorescence intensity of the donor sample, and *F_DA_* is the fluorescence intensity of the sample containing donor and acceptor GpA TM domains at equal concentrations. The energy transfer of sequence-specific TM helix dimerization, *E_D_*, can be expressed as:
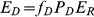
(2)where *f*_D_ is the fraction of dimeric TM helices, *P*_D_ is the probability of donor quenching when the peptides form a dimer, and *E*_R_ is the energy transfer in the dimer.

The probability *P*_D_ of donor quenching depends on the molar ratio of the acceptor peptides 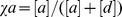, where [a] and [d] are the concentrations of acceptor and donor peptides, respectively. If the distance of the fluorophores in the dimer is much smaller than the Förster radius, as is the case here, *E_R_* can be taken as unity.

The fraction of dimers can be written as 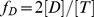, where [D] is the concentration of dimeric peptides and [T] the total peptide concentration. Hence, the dimer concentration [D] can be calculated as [24], [25], [29]:
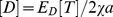
(3)


The dissociation constant, *K*_D_, is given by
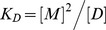
(4)where the monomer concentration is 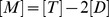.

### Circular dichroism spectroscopy

CD spectra were recorded on a Jasco J-815 spectropolarimeter at 25°C in a step-scan mode using 0.1-cm path length quartz cells from Hellma (Mülheim, Germany). The concentration of unlabeled GpA peptide was 5 µM. Data points were collected at a resolution of 1 nm, an integration time of 1 s, and a bandwidth of 1 nm. Each shown spectrum results from at least three averaged scans from which buffer scans were subtracted. The measured ellipticity θ was converted to molar ellipticity [θ] by:
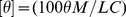
(5)where *M* is the molar mass, *L* the path length, and *C* the peptide concentration.

The GpA TM domain was reconstituted in 10 mM phosphate buffer (pH 7.4) containing A8-35 as well as in several solvents containing SDS, DDM, or pure TFE, which are known to stabilize α-helical structures. Prior to CD measurements, samples were treated in the same way as described above for FRET measurements. Secondary structure contents were estimated with the DICHROWEB software [30], [31] using both soluble and TM proteins as reference datasets (CDSSTR method, reference set 7).

### Isothermal titration calorimetry (ITC)

ITC experiments were performed on a VP-ITC (GE Healthcare) at 25°C in 10 mM phosphate buffer (150 mM NaCl, pH 7.4). For demicellization experiments, 5-µL aliquots containing both, SDS at a concentration well above its CMC (10-13 mM) as well as A8-35 at concentrations of 5-140 µM, were injected into the sample cell containing the same amphipol concentration but no SDS. Time spans between injections were chosen long enough to allow for complete re-equilibration. Baseline subtraction and peak integration were accomplished using NITPIC [32], and the resulting isotherms were analyzed by nonlinear least-squares fitting in a spreadsheet program [33].

## Results and Discussion

### GpA TM helix dimerization in A8-35

**Figure 1:**
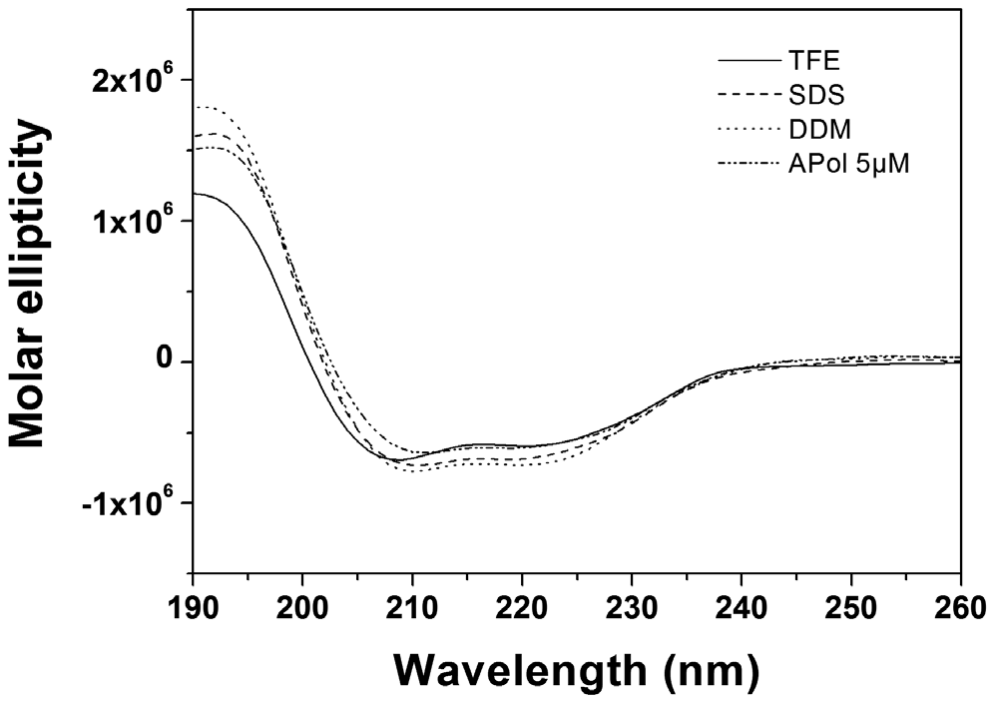
Far-UV CD spectra of the GpA TM domain. GpA TM domain (5 µM) solubilized in 5 µM APol. As controls, GpA TM domain was solubilized in TFE, SDS, or DDM, which are known to support the formation of α-helical structures.

First, we determined the secondary structure of GpA TM peptides solubilized in A8-35 by far-UV CD spectroscopy to ensure that the GpA TM peptides were fully solubilized and adopted the expected α-helical structure in the APol environment (Figure 1). As a control, the secondary structure of the GpA TM domain was additionally analyzed in trifluoroethanol (TFE), 5 mM SDS, or 5 mM DDM, since it has been shown that α-helical structures are stabilized in these solvents [24], [26], [34]. The CD spectra demonstrate that a maximum at 190 nm and double minima at 209 nm and 222 nm are retained in 5 µM A8-35, which are characteristic of α-helical structures. Higher APol concentrations did not significantly affect the peptide's secondary structure. Estimation of the secondary structure contents suggested an α-helix content of 67% in 5 µM A8-35, compared to 62% in TFE, 76% in SDS or 79% in DDM. Thus, the structural hallmarks of the GpA TM helix are largely preserved in A8-35 particles, and therefore the effects of increasing A8-35 concentrations on the stability of the GpA TM helix dimer were studied in subsequent experiments.

**Figure 2:**
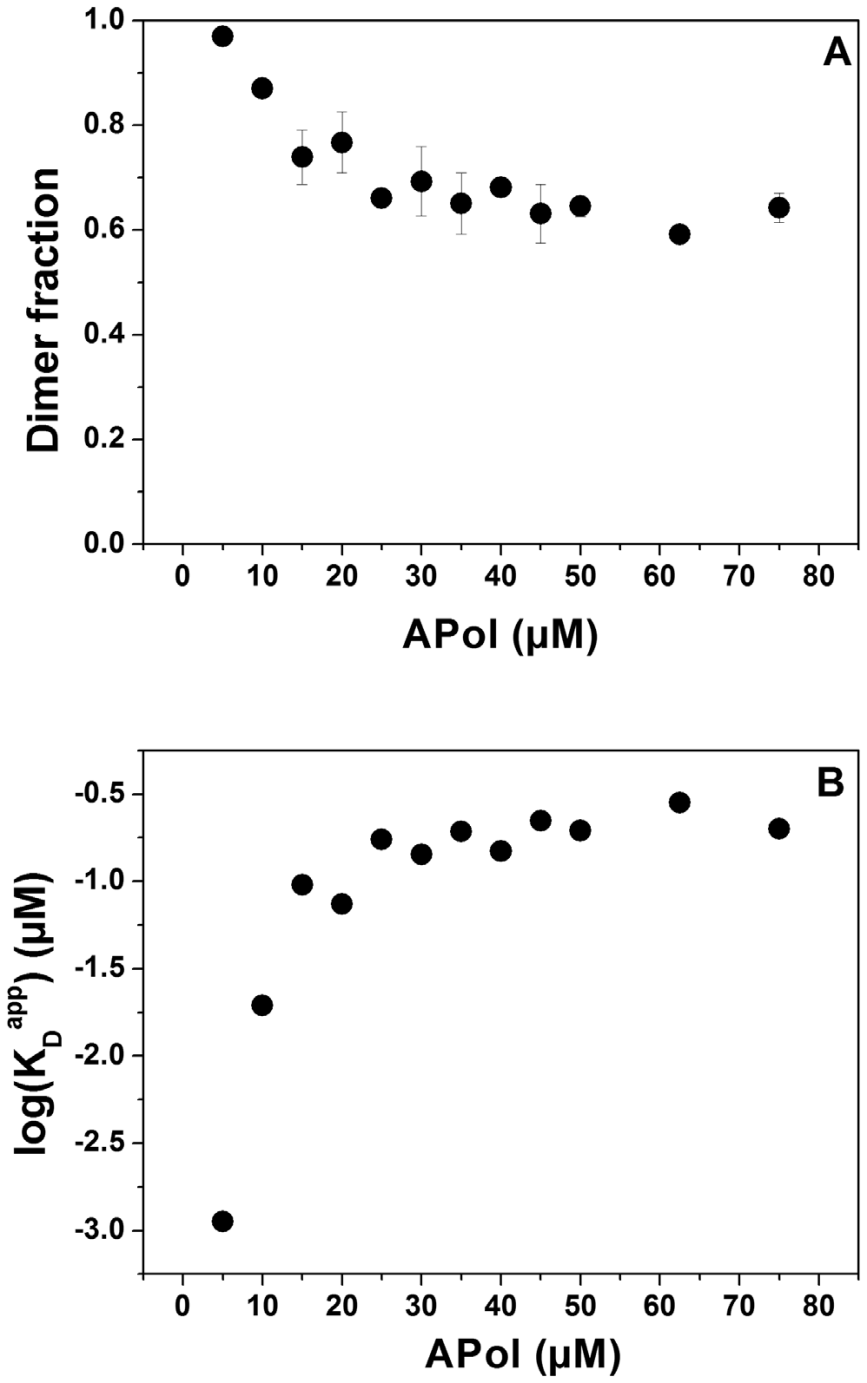
Association of the GpA TM domain in APol. FRET measurements (n = 3) were performed at increasing APol concentrations ranging from 5 to 75 µM, corresponding to APol/peptide ratios of 10∶1 to 150∶1. (A) Dimer fractions of the GpA TM domain determined by FRET efficiencies of the fluorescence emissions' spectra plotted against APol concentration (see eq. 3). (B) Logarithm of the apparent GpA TM dissociation constants at increasing APol concentration (see eq. 4).

To determine the thermodynamic stability of the GpA TM helix dimer, FRET measurements were performed in APol A8-35. To do so, the GpA TM peptides were chemically labeled at the N-terminus with either fluorescein (FL) or carboxytetratmethyl-rhodamine (TAMRA). Figure S1 shows the emission and excitation spectra of the labelled peptides solubilized in A8-35. The emission spectrum of the fluorescein-labelled peptide substantially overlaps with the excitation spectrum of the TAMRA-labelled peptide. Therefore, energy transfer is a direct measure of the fraction of dimeric peptide species in the sample [24], [25], [28], [29]. In the first series of experiments, the total peptide concentration of the FRET pair was kept constant at 0.5 µM, while the A8-35 concentration was increased from 5 to 75 µM, which corresponds to APol-to-peptide ratios of 10∶1 to 150∶1. As can be seen in Figure 2A, the fraction dimeric GpA decreased with increasing A8-35 concentrations. Almost all peptides were dimeric at low A8-35 concentrations, whereas the dimer fraction dropped to a value of about 0.65 at 25 µM A8-35 and thereafter decreased only faintly with increasing polymer concentrations. This indicates that the GpA TM helix dimer is rather stable even at higher A8-35 concentrations and only very high polymer concentrations will dissociate the dimer completely. For comparison, detergent micelles destabilize the GpA dimer to a larger extent, even if the detergent concentration exceeds the CMC only slightly [24], [26]-[28]. However, the extent of GpA dimerization in detergents depends severely on the head group, the chain length and the concentration of the particular detergent. In general, APol A8-35 appears to stabilize the GpA TM domain dimer rather well, as the dissociation constants are below 0.3 µM in the tested A8-35 concentration range (Figure 2B), and even relatively higher A8-35 concentrations do not seem to fully dissociate the GpA dimer, in contrast to detergents. While at low detergent concentrations the dissociation constants of the GpA TM dimer in several tested detergents (SDS, DDMAB, DPC, lyso-PC) are in the same range as in APol (≤0.5 µM), they increase significantly, compared to A8-35, upon further addition of detergent until the dimer is completely dissociated [26]-[28].

Detergents affect helix association in a rather complex way: In the first place, simple dilution of the peptide in the micellar phase entropically promotes dissociation with increasing detergent concentrations [27]. Additionally, however, opposing enthalpic and entropic effects, which depend on the nature of the detergent and are generally poorly understood, may counteract dilution by enhancing helix association with increasing detergent concentrations. The high stability of the GpA TM helix dimer in A8-35 might be explained by (i) a poor ability of APols to compete with protein/protein interactions, (ii) the reduced size of the hydrophobic sink compared to detergents [35], or (iii) a dampening effect of potential conformational fluctuations due to the viscosity of the polymer backbone [3], [36].

**Figure 3:**
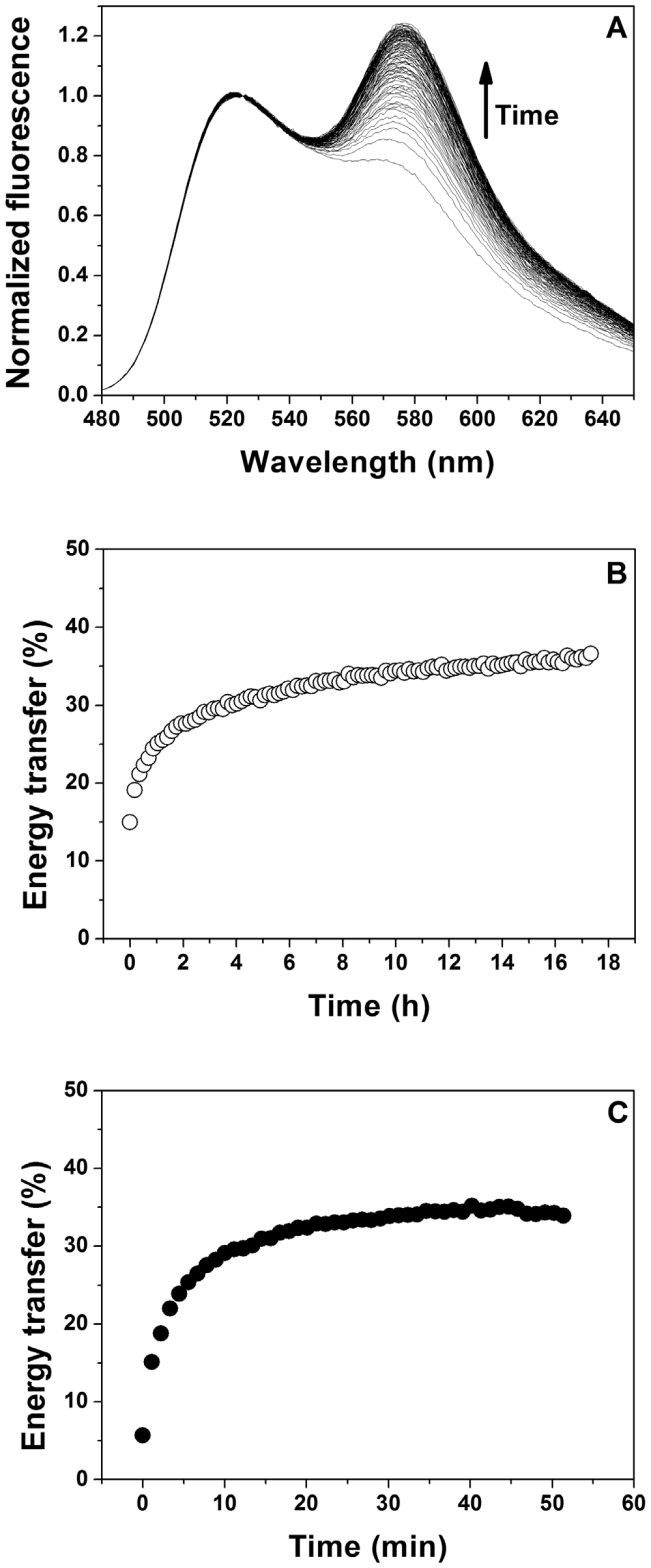
Kinetics of the exchange of GpA TM peptides between APol aggregates. Donor- and acceptor-labelled GpA TM (each 0.25 µM) domains were separately solubilized in 20 µM APol at a final polymer/peptide ratio of 40∶1. (A) After incubation at 37°C, donor and acceptor were mixed, and emission spectra (normalized at 525 nm) were recorded every 10 min over a time period of 17 h at 25°C. (B) The energy transfer increased over hours to the level determined by steady-state FRET measurements due to mixing of donor- and acceptor-labelled peptides. (C) Energy transfer change due to peptide exchange in 5 mM DDM micelles was completed after some dozen minutes.

To investigate the effect of A8-35 on the GpA dimer stability in greater detail, we next performed kinetic measurements and determined the exchange rates of GpA TM helices between various polymer particles after reconstitution. Upon addition of an excess of a competing surfactant, such as free APols, detergents or phospholipids, MPs can be released from preformed complexes with APols, although at very slow dissociation rates, and *e.g.* integrate into detergent micelles [3], [37]-[39]. To test this, donor- and acceptor-labeled GpA peptides were individually solubilized in 20 µM A8-35 and incubated at 37°C for at least 2 h. Next, A8-35-solubilized donor and acceptor peptides were mixed in a 1∶1 ratio, and fluorescence emission spectra were recorded every 10 min for about 17 h (Figure 3A). As a control necessary for calculation of energy transfer, this measurement was also performed with solely the donor sample, for which no changes in fluorescence intensity were observed. Figure 3B shows that 17 h after mixing the two differently labelled GpA TM helices, the energy transfer increased up to ∼35%, which corresponds to a dimer fraction of ∼0.7, as already observed before in the steady-state measurements (Figure 2A). To compare the exchange rates measured in APols with rates determined in micellar environments, the same experiment was performed in 5 mM DDM micelles (Figure 3C). While the exchange of donor- and acceptor-labeled GpA TM peptides suspended in A8-35 proceeded for several hours, the exchange of labeled peptides between micelles was already complete after several minutes. Proper fits of the measured kinetics were obtained with a double-exponential equation yielding rate constants of 2.2 h^-1^ (amplitude 8.7) and 0.17 h^-1^ (amplitude 12.4) for A8-35, and 41.5 h^-1^ (amplitude 13.3) and 5.8 h^-1^ (amplitude 15.6) for DDM micelles.

**Figure 4:**
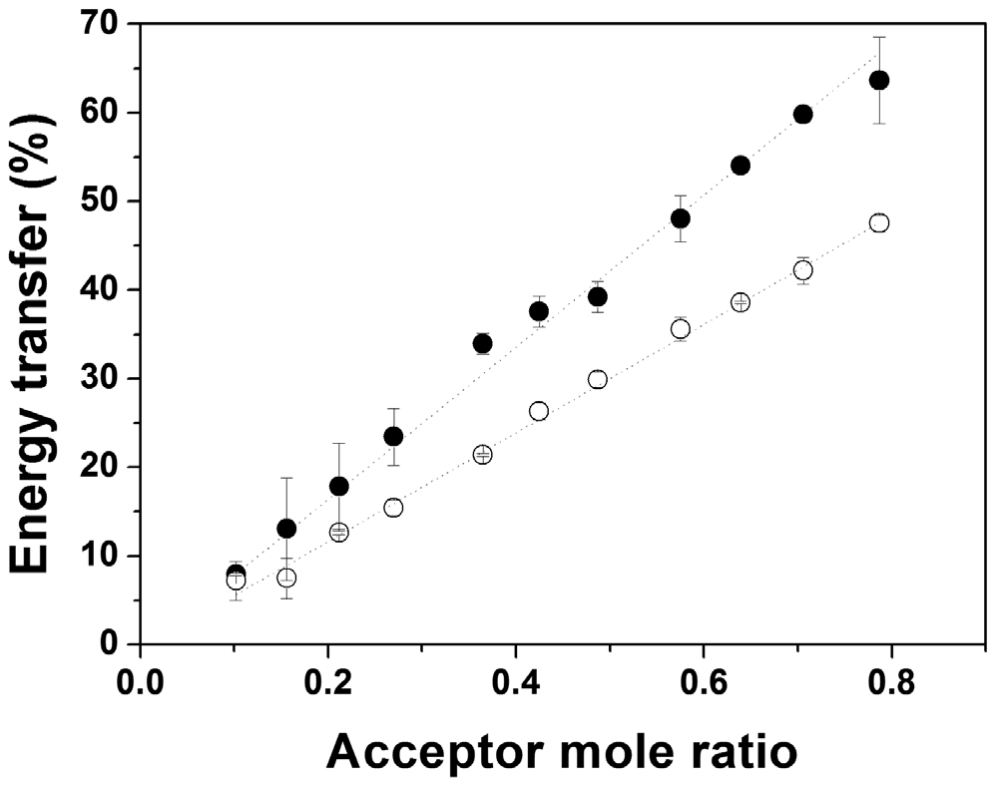
Stoichiometry of GpA TM domain association. Energy transfer efficiency as a function of acceptor mole fraction in 5 µM (•) and 50 µM (○) APol (n = 2). Linear dependence of the energy transfer on the acceptor mole ratio demonstrates exclusive dimer formation of the GpA TM domain in APol.

Besides formation of GpA dimers, the recorded energy transfer might, in principle, also originate from formation of higher-ordered oligomeric structures, such as trimers or tetramers, or even from unspecific peptide aggregation. Such a possibility can be excluded by measuring energy transfer at different acceptor mole ratios (*χ*_a_) while keeping the total peptide concentration constant. If the energy transfer linearly depends on *χ*_a_, only the formation of dimers will contribute to the energy transfer measured [23]. As shown in Figure 4, at 5 µM and 50 µM A8-35, the energy transfer linearly depends on *χ*_a_, indicating exclusive formation of GpA dimers in APol A8-35.

### GpA TM domain dimerization in mixed APol/SDS micelles

**Figure 5:**
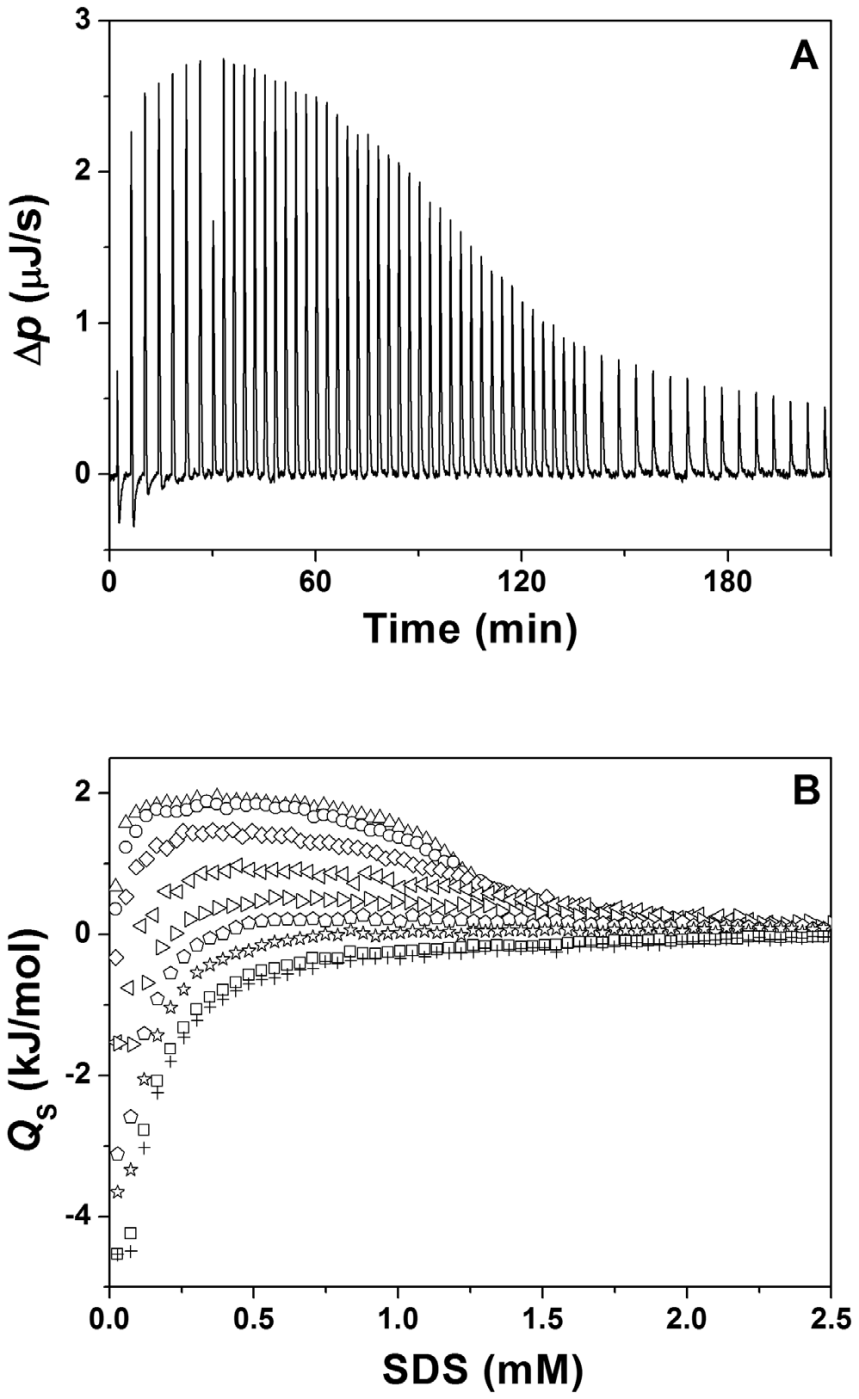
Interactions of SDS with A8-35 monitored by isothermal titration calorimetry. Demicellization of SDS in the presence of various A8-35 concentrations: (A) Differential heating power, Δ*p*, versus time, monitored during the titration of 10 mM SDS and 20 µM A8-35 into 20 µM A8-35. (B) Normalized heats of reaction, *Q*_S_, versus SDS concentration in the cell, [SDS], resulting from the dilution of micellar SDS solutions (10-13 mM) in the presence of A8-35 at A8-35 concentrations of 5 µM (△), 10 µM (O), 20 µM (◊), 40 µM (◃), 60 µM (▹), 80 µM (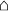), 100 µM (☆), 120 µM (□), and 140 µM (+).

Thermal denaturation of MPs often leads to irreversible aggregation of the MP, and guanidine hydrochloride and urea, which are frequently used in unfolding studies involving soluble proteins, do typically not denature the TM regions of MPs. This is why the thermodynamic stability of integral MPs within membrane-mimetic systems is often assessed by titrating increasing concentrations of the anionic detergent SDS to MPs dissolved in a mild detergent, such as DDM [24], [40]-[44]. SDS is able to form mixed micelles with other detergents typically used for MP solubilization. In SDS-containing mixed micelles, a membrane mimetic environment is maintained and the secondary structure of the MPs TM regions is barely affected by SDS-induced protein unfolding [40]. However, it is worth mentioning that the term “unfolding” in this system in fact describes dissociation of TM helices rather than unfolding of individual TM α-helices [24]. Since mixed DDM/SDS micelles dissociate the GpA TM helix dimer almost completely [24], a similar approach based on the denaturing effect of SDS might be useful for investigating the thermodynamic stability of the GpA TM helix dimer in APol solutions. Furthermore, when amphipols are used *in vitro*, detergents are still needed to extract MPs from their natural membrane, and, therefore intermediate states of MPs in mixed APol/detergent micelles are present during the experimental procedure. Preserving the MPs' structure and activity during this process is fundamental for subsequent experiments. To shed more light on the interactions between A8-35 and the anionic detergent SDS, we performed a series of ITC experiments (Figure 5), in which SDS solutions at concentrations above the CMC (10-13 mM) were diluted into buffer in the presence of increasing A8-35 concentrations. At low APol concentrations, the isotherms thus obtained resembled those measured in SDS demicellization experiments in the absence of APol [45]. However, with increasing A8-35 concentrations, the isotherms became shallower and even changed sign, similarly to what has previously been observed for the interactions of the non-ionic detergent octylglucoside with A8-35 and other amphipols [13]. However, while the latter have been found to follow nearly ideal mixing behavior [13], the interactions of SDS with A8-35 follows a more complex pattern (Figure 5). Although the reaction heats could be fitted on the basis of the regular mixing model at intermediate SDS contents in the mixed aggregates [13], such fits yielded very high nonideality parameters of 3–4 *RT*, which is incompatible with the near-ideal mixing assumption inherent in this model. At both lower and higher SDS concentrations, the isotherms could not at all be analyzed in terms of simple mixing models, which is most likely due to a charge repulsion between free SDS monomers and mixed APol/SDS aggregates or due to the changes in pH at the surface of the mixed aggregate [46], [47]. Thus, care should be taken in interpreting SDS titration data involving anionic APols at a quantitative level, which is why we restrict ourselves to qualitative considerations in the following.

**Figure 6:**
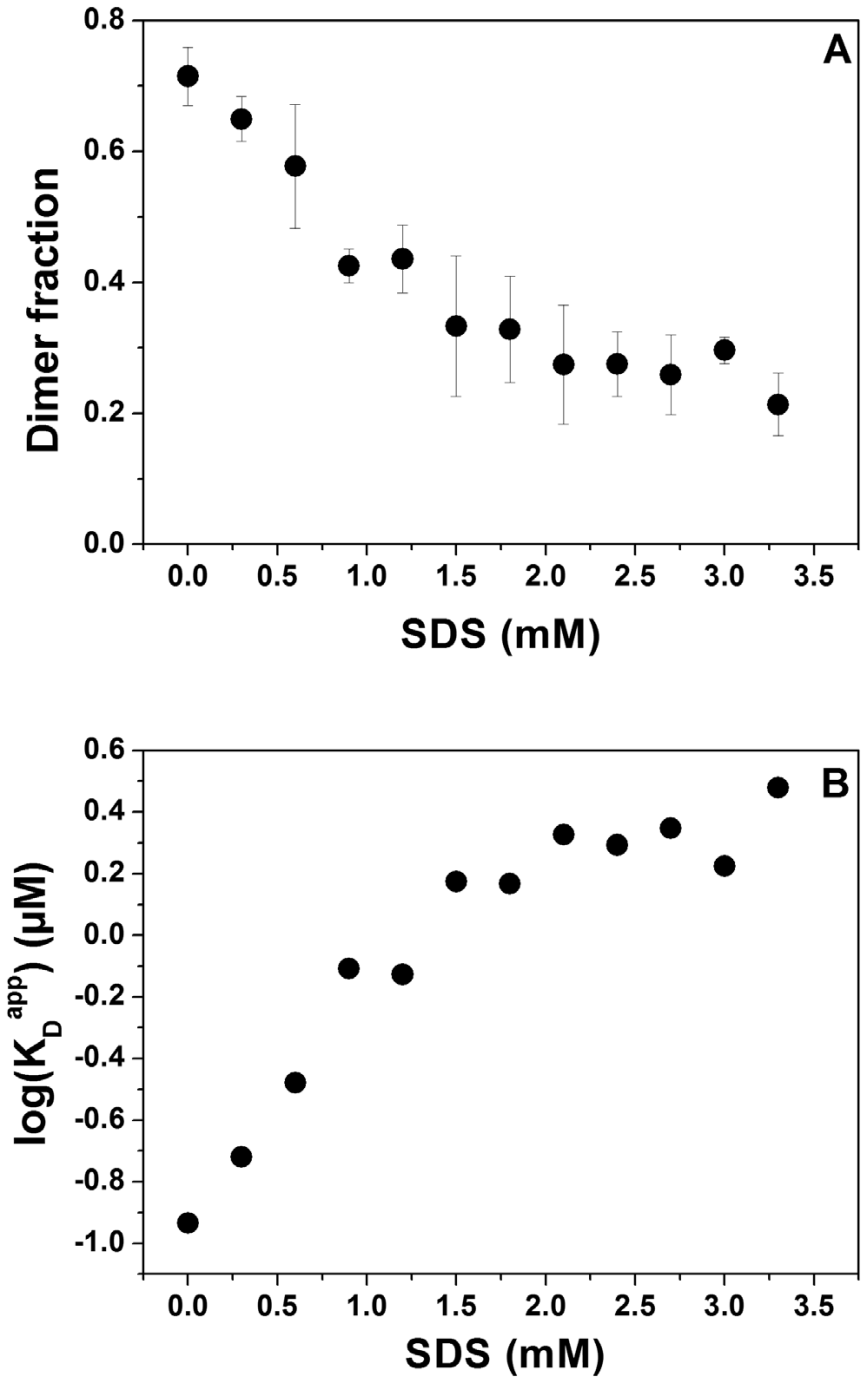
Dissociation of the GpA TM domain in APol upon addition of SDS. Steady-state FRET measurements (n = 3) were performed at a fixed polymer (20 µM) to peptide (0.5 µM) ratio (40∶1). SDS was added to preformed APol/GpA complexes, and emission spectra were recorded at 25°C after incubation at 37°C. (A) Dimer fractions of the GpA TM domain determined by FRET efficiencies of the fluorescence emissions spectra plotted against SDS concentration (see eq. 3). (B) Logarithm of the apparent GpA TM dissociation constants at increasing SDS concentration (see eq. 4).

To address the question of how SDS/APol aggregates/micelles affect TM helix-helix association and hence the stability of α-helical MPs, FRET efficiencies were determined at increasing SDS concentrations, while the concentrations of the GpA TM domain (0.5 µM) and APol A8-35 (20 µM) were kept constant. Under these conditions, the fraction of dimeric GpA decreases from 0.7 in the absence of SDS to about 0.3 at SDS concentrations exceeding 1.5 mM (Figure 6A), corresponding to an increase in the dissociation constant by about one order of magnitude (Figure 6B). This indicates a strong destabilization of the GpA dimer after addition of SDS, resulting in dissociation of GpA TM dimers in mixed A8-35/SDS aggregates. As previously observed in mixed detergent micelles [24], addition of SDS to A8-35-solubilized GpA destabilizes the GpA dimer. However, in contrast to DDM/SDS mixed micelles, in which the GpA dimer is completely unfolded [24], this was not observed in case of APol/SDS mixed micelles, at least not in the analyzed concentration range (Figure 6). Thus, SDS appears to be less denaturing when a MP is solubilized in A8-35 compared to classical detergents. However, a direct comparison of the SDS denaturation data in DDM vs. A8-35 is complicated, as the chemical nature of DDM and A8-35 are very different and usually SDS mole fractions are used for plotting the denaturation process. Using mole fractions is not very helpful in case of APol/SDS, as the high molecular mass polymer carries multiple hydrophobic tails and much lower APol concentrations are needed to solubilize the TM helix compared to DDM. A8-35 aggregates can also not be directly compared with detergent molecules and micellar structures. Furthermore, the mixing behavior of SDS and A8-35 is quite complex, as the ITC data indicate (Figure 5). In addition, the degree of non-ideality in DDM/SDS mixing is also very complex and varies over experimental conditions. Therefore, heterogeneities in the mixed micelles/aggregates cannot be excluded [48], and the spatial structure of APol/SDS assemblies is likely to be very different from a typical micelle due to the long polymer backbone.

**Figure 7:**
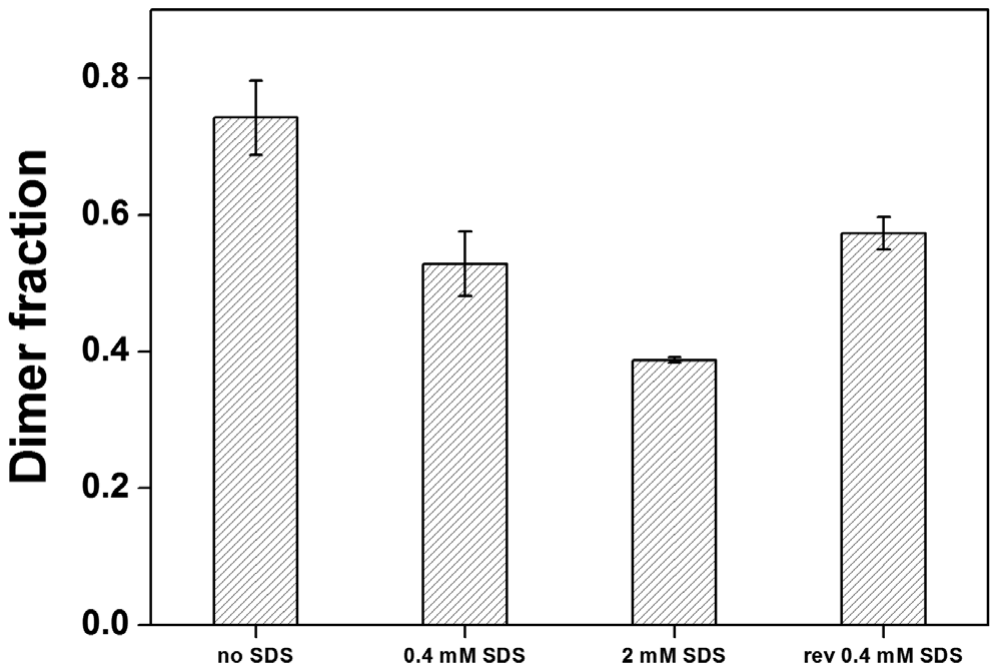
Reversibility of GpA dimer dissociation by SDS addition to APol/GpA complexes. Dimer fractions shown without SDS and with 0.4 mM and 2 mM SDS added. The 2 mM SDS sample was then diluted to 0.4 mM SDS to demonstrate the reversibility of SDS-mediated dimer dissociation. The fraction dimer at 0.4 mM SDS after dilution from higher SDS concentrations is comparable to that observed at 0.4 mM SDS (n = 3).

To assess the reversibility of the observed changes in dimer stability, FRET was measured at 2 mM SDS and 20 µM APol, both prior to and following dilution of the sample with another sample, containing no SDS but the same concentrations of peptide and APol (Figure 7). Thereby, the SDS concentration was diluted below its CMC (to 0.4 mM), while the concentrations of peptide and APol were kept constant. The fraction of dimeric GpA observed after dilution agrees with the value determined at 0.4 mM SDS (Figure 7) and the titration experiments (Figure 6A), indicating that dissociation and association of the GpA TM helix in APol A8-35, caused by SDS addition and removal, respectively, are fully reversible. Thus, addition of SDS to APol-reconstituted GpA TM peptides below the CMC of SDS appears to dramatically destabilize the GpA dimer, which might be caused by the altered structure of the APol aggregate, and thus the altered local environment of the GpA TM helix dimer. Furthermore, addition of extra negative charges might result in increased electrostatic interactions of a stretch of positive charges in the GpA juxtamembrane region, which could destabilize the helix dimer [49], or in an more acidic local pH at the surface of the mixed aggregate [46]. Nevertheless, since mixing of APol and SDS is strongly non-ideal, further analyses are needed for a more detailed, quantitative treatment.

## Conclusions

In recent studies, the impact of A8-35 on the stability and activity of selected TM proteins has already been addressed. However, in the preceding studies, polytopic TM proteins have been analyzed, where multiple short- and long-range interactions might stabilize the TM protein structure. Here we showed that trapping a MP in APol A8-35 aggregates does not prevent the sequence-specific interaction of a single TM helix. Refolding and oligomerization of the GpA TM helix dimer in APol A8-35 are possible, avoiding the use of large amounts of detergent. Addition of SDS to APol-trapped GpA weakens the dimerization of the GpA TM helix, as observed in DDM/SDS mixed micelles before, although the effect appears to be less denaturating and low SDS concentrations are tolerated with respect to the formation of tertiary contacts between the helices. The use of APols therefore facilitates the use of very low surfactant concentrations to analyze the folding of a MP under milder conditions. APol/SDS mixed micelles might be useful as a tool for structure-function analyses, since the oligomeric state and the activity of a MP can be tuned judiciously. This can turn out to be important when structural dynamics is needed for protein activity, as the stabilizing environment of APols might inhibit MP function. Thus, addition of detergents could loosen up the surrounding polymer belt, allowing structural dynamics and functionality of an APol-solubilized MP.

## Supporting Information

Figure S1 **Excitation (solid lines) and emission spectra (dashed lines) of Fl (donor)- and TAMRA (acceptor)-labeled GpA peptides.** (A) Fl-GpA, excitation 439 nm, emission 530 nm. (B) TAMRA-GpA, excitation 530 nm, emission 590 nm. Spectra are normalized. (C) Fluorescence emission spectra of donor- and acceptor-labeled peptides (dashed line) as well as control samples containing only donor-labeled peptides (solid line) and only acceptor-labeled peptides (dotted line) upon excitation at 439 nm. Spectra were recorded in 10 mM HEPES buffer containing 150 mM NaCl and 20 µM APol at pH 7.4. Exitation spectra were measured with both slits set at 2 nm. Emission spectra were measured with both slits set at 3 nm.
(TIF)
