# Supplementary figures and images for: Sequence-Specific Dimerization of a Transmembrane Helix in Amphipol A8-35
Source: PLoS One. 2014 Oct 27;9(10):e110970. doi: 10.1371/journal.pone.0110970 (PMC4210147; doi:10.1371/journal.pone.0110970)

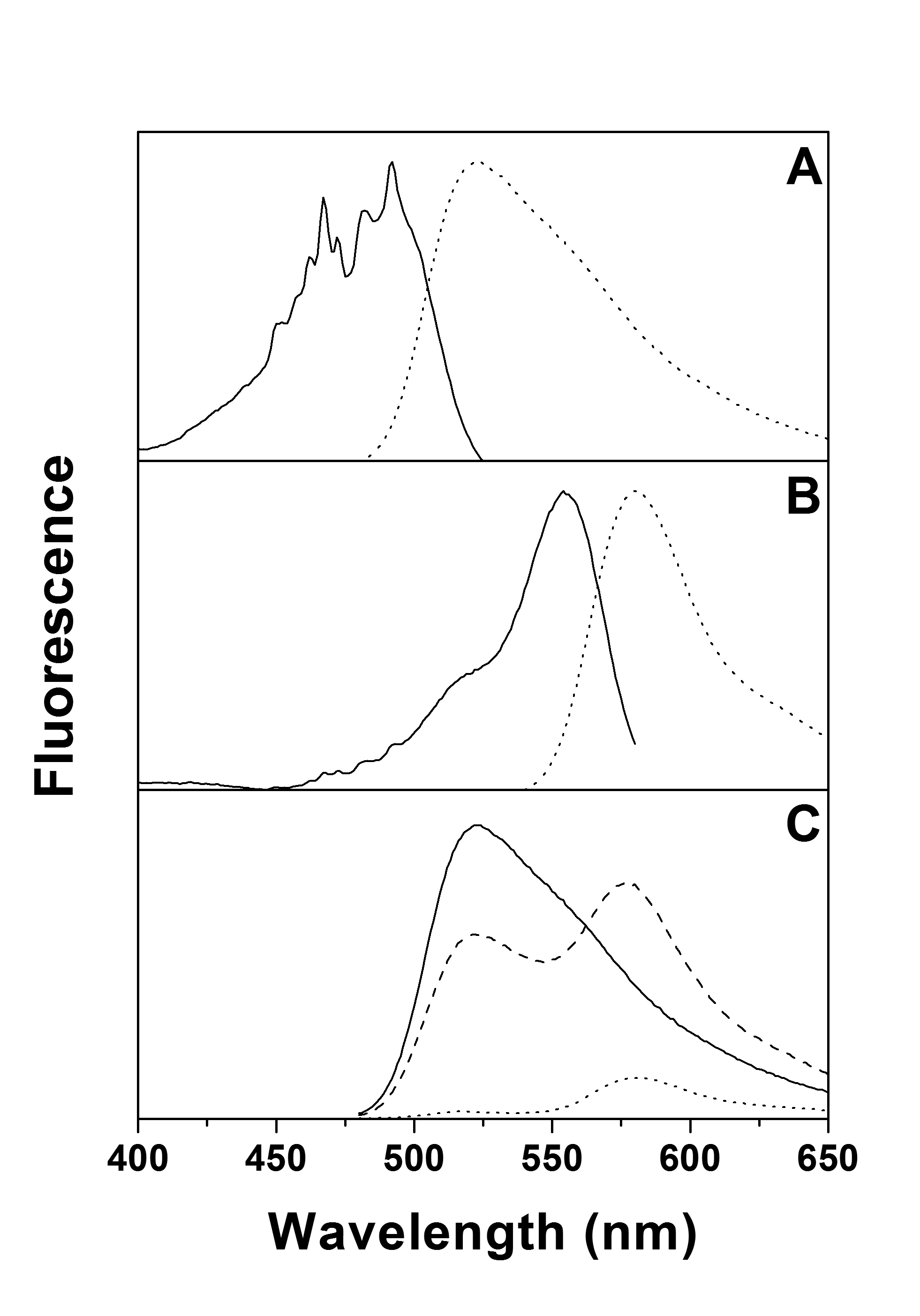

Supplement: Figure S1 — Excitation (solid lines) and emission spectra (dashed lines) of Fl (donor)- and TAMRA (acceptor)-labeled GpA peptides. (A) Fl-GpA, excitation 439 nm, emission 530 nm. (B) TAMRA-GpA, excitation 530 nm, emission 590 nm. Spectra are normalized. (C) Fluorescence emission spectra of donor- and acceptor-labeled peptides (dashed line) as well as control samples containing only donor-labeled peptides (solid line) and only acceptor-labeled peptides (dotted line) upon excitation at 439 nm. Spectra were recorded in 10 mM HEPES buffer containing 150 mM NaCl and 20 µM APol at pH 7.4. Exitation spectra were measured with both slits set at 2 nm. Emission spectra were measured with both slits set at 3 nm. (TIF) [file pone.0110970.s001.tif]
